# OLDER AND YOUNGER AS OTHER: OBSTACLES TO AGE-FRIENDLINESS ON CAMPUS

**DOI:** 10.1093/geroni/igae098.2942

**Published:** 2024-12-31

**Authors:** Summer Roberts, Cindy Lahar

**Affiliations:** University of South Caroline Beaufort, Bluffton, South Carolina, United States; University of South Carolina Beaufort, Bluffton, South Carolina, United States

## Abstract

The University of South Carolina Beaufort (USCB) drafted an endorsement of the Age-Friendly University Principles in 2020; however, this effort has yet to advance beyond the working group. USCB, whose main campus is situated intentionally between two retirement communities, and with a large and active Osher Lifelong Learning Institute (OLLI) offering courses across three campuses, clearly reflected the principles on paper. Over the next three years, students in a sociology research seminar studied, at the request of the county, the needs of the region’s growing and diverse older adult population. Yet, these data collection efforts reflected limited exposure to people of older ages, resulted in primarily white and high SES samples, and separated USCB from community efforts to support older adults. Additionally, anecdotal accounts of staff and students commonly note that visitors to campus disparage traditional college-aged students. As a result, focus has shifted to more fully integrating students in shaping age-inclusive campus culture beyond coexisting age-segregated environments. This presentation explores efforts through three courses to develop an action plan. Qualitative analysis of papers from gerontology classes identifies the attitudes of students surrounding their relationship with older adults in the community and on campus, particularly highlighting the consistent use of othering and ageism in talking about those of different chronological ages. Students’ on-going research of fellow students’ and community members’ perceptions of age-friendliness provides the basis for promoting intergenerational solidarity at USCB.

